# NR4A1 suppresses pyroptosis by transcriptionally inhibiting NLRP3 and IL‐1β and co‐localizing with NLRP3 in trans‐Golgi to alleviate pathogenic bacteria‐induced colitis

**DOI:** 10.1002/ctm2.639

**Published:** 2021-12-19

**Authors:** Zhao Deng, Zhipeng Yang, Chenbin Cui, Hongkui Wei, Lijia Wang, Dean Tian, Fang Xiao, Jian Peng

**Affiliations:** ^1^ Department of Animal Nutrition and Feed Science, College of Animal Science and Technology Huazhong Agricultural University Wuhan 430070 P. R. China; ^2^ Department of Gastroenterology, Tongji Hospital of Tongji Medical College Huazhong University of Science and Technology Wuhan Hubei Province 430030 China; ^3^ The Cooperative Innovation Center for Sustainable Pig Production Wuhan Hubei 430070 China

Dear Editor,

We present an effective target to treat colitis would be achieved through the transcriptional regulation and trans‐Golgi translocation of NR4A1, so as to inhibit NLR family, pyrin domain containing 3 (NLRP3) inflammasome. Inflammatory bowel disease (IBD) is a chronic and intractable digestive tract inflammatory disease that affects the millions of people; blocking immune responses and inflammatory cytokines have become effective strategies for treating colitis. NR4A1 (also known as TR3, NGFIB or Nur77), a member of the nuclear receptor NR4A family, exerts a protective role in colitis,^1^, ^2^ however, its effect and mechanism on pyroptosis or NLRP3 inflammasome is still unclear.

In current study, NR4A1 expression is significantly increased in colon tissues from *C. rodentium* (*Citrobacter rodentium*)‐induced mice colitis (Figure 1A,B). And *NR4A1* deficiency increases *C. rodentium*‐induced mortality, weight loss, colon shorten, colon mucosal damage and the colonization of *C. rodentium* (Figure 1C‐F and Figure S1B‐F). For pyroptosis and inflammasome, *NR4A1* deficiency increases the level of lactate dehydrogenase (LDH), interleukin‐1β (IL‐1β), IL‐18, caspase‐1 p20 and the cleavage of gasdermin D (GSDMD) in serum or colon (Figure 1G‐L and Figure S1G,H). These results indicated that *NR4A1* deficiency exacerbates inflammasome activation and pyroptosis in vivo.

**FIGURE 1:**
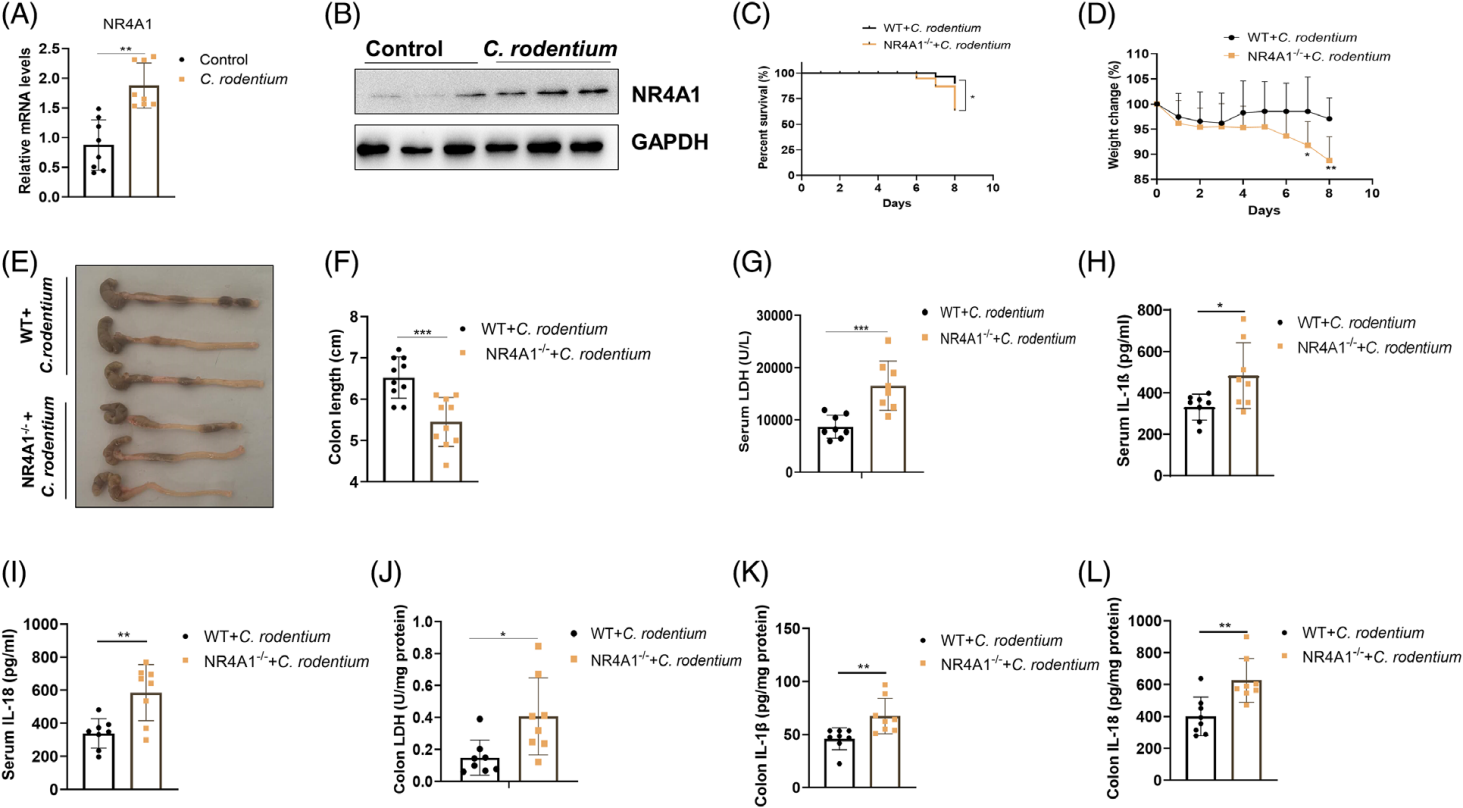
NR4A1 protects against *C*. *rodentium*‐induced colitis. Wild type (WT) mice (*n* = 8/group) were gavaged with *C*. *redentium* (5 × 10^9^ CFU) at day 1, then where sacrificed at day 8 after colitis induction. (A and B) quantitative polymerase chain reaction and immunoblot analysis of NR4A1 expression in colon tissues. WT and *NR4A1* ^‐/‐^ mice (*n* = 10/group) were gavaged with *C*. *rodentium* (5 × 10^9^ CFU) at day 1, then were sacrificed at day 8 after colitis induction. (C) Survival percentage of mice after *C. rodentium* treatment. (D) Weight change of mice during the experiment. (E and F) Images and statistical analysis of colon length. (G‐I) Production of LDH, IL‐1β and IL‐18 in mice serum. (J‐L) Production of LDH, IL‐1β and IL‐18 in mice colon tissue. Values are expressed as mean ± SD, ^*^ *p *< 0.05

To investigate the role of NR4A1 in inflammasome activation, we isolated bone marrow‐derived macrophages (BMDMs) from WT mice and *NR4A1^−/−^* mice. For canonical inflammasome, the results showed that NR4A1 only inhibits NLRP3 inflammasome, without affecting NLRC4 or AIM2 inflammasome (Figure S2A,B). NR4A1 expression is increased after the first and the second stimulation of NLRP3 inflammasome (Figure S2C). And *NR4A1* deficiency augments Nigericin‐induced LDH, IL‐1β, IL‐18 and caspase‐1 cleavage (Figure 2A‐E and Figure S2E,F). Except Nigericin, NLRP3 inflammasome is reported to be activated by other stimulants^3^; *NR4A1* deficiency also significantly increases adenosine triphosphate (ATP) and monosod ium urate‐induced NLRP3 inflammasome activation (Figure S2A,B). For non‐canonical NLRP3 inflammasome activation, NR4A1 expression is increased after treatment, and *NR4A1* deficiency increases cytosolic lipopolysaccharide (cLPS)‐induced LDH, IL‐1β and IL‐18 production (Figure S3A‐D); WT and *NR4A1^−/−^* mice were injected with lipopolysaccharide (LPS) to induce non‐canonical NLRP3 inflammasome activation, *NR4A1* deficiency exacerbates LPS‐induced jejunum pathological injury, concentration of IL‐1β and IL‐18 in serum (Figure S3G‐I). During NLRP3 inflammasome activation, ASC and NLRP3 oligomerization are acritical steps for the subsequent caspase‐1 activation.^3^, ^4^, ^5^, ^6^ *NR4A1* deficiency aggravates Nigericin‐induced amounts of ASC specks, and the interaction between NLRP3 and ASC in BMDMs (Figure 2F and Figure S4). Meanwhile, overexpression of NR4A1 inhibits canonical and non‐canonical NLRP3 inflammasome activation in THP‐1 and BMDMs (Figure S2D,G and Figure S3E,F). Taken together, these results provided sound evidence that NR4A1 would inhibit the activation and assembly of NLRP3 inflammasome.

**FIGURE 2:**
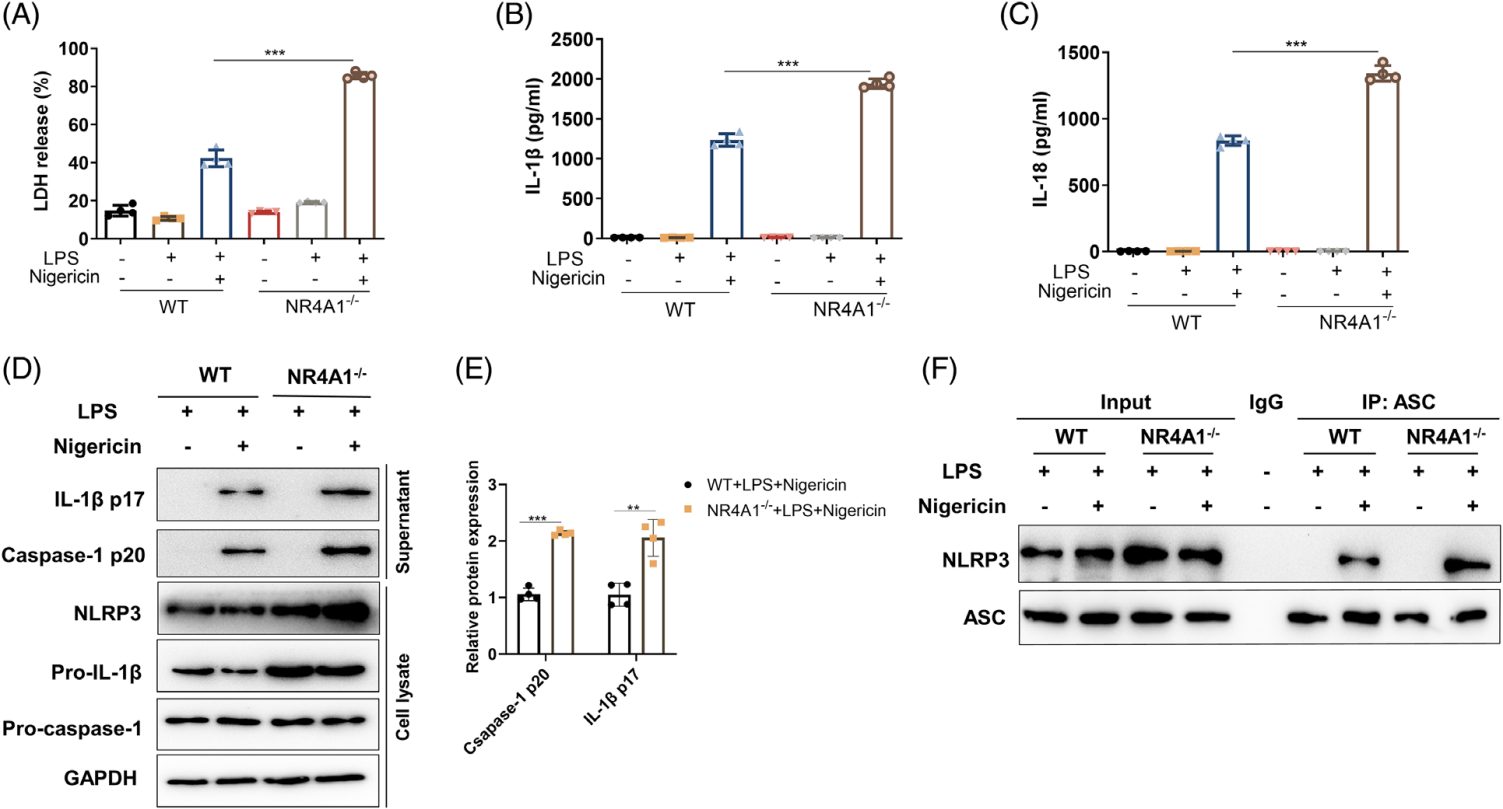
NR4A1 dificiency aggravates canonical NLRP3 inflammasome activation and assembly in macrophages. Canonical NLRP3 inflammasome activation, BMDMs were primed for 4 h with LPS (200 ng/ml) and then stimulated with Nigericin (10 μM) for 40 min. (A‐C) Supernatants were analyzed for LDL, IL‐1β and IL‐18 release. (D and E) Immunoblot analysis of IL‐1β p17 and caspase‐1 p20 in supernatants, and immunoblot analysis of NLRP3, pro‐caspase‐1 and pro‐IL‐1β in cell lysates. (F) IP and immunoblot analysis of the interaction of endogenous NLRP3 and ASC. Values are expressed as mean ± SD, *p < 0.05, three independent experiments

The activation of NLRP3 inflammasome requires two sequential steps, that is, priming and activation, and the key priming event is the NF‐κB pathway‐mediated transcription of pro‐IL‐1β and NLRP3.^3^ *NR4A1* deficiency increases the expression of NLRP3 and pro‐IL‐1β in macrophages and colon tissues (Figure 3A,B, Figures S2E and S5A,B). As a transcription factor, sequence analysis of the mouse NLRP3 and IL‐1β promoter reveals that either of which contains the conserved potential monomeric NR4A1 binding sites NGFIB response element (NBREs) (Table S1). Through luciferase reporter assays, promoter deletion and deletion experiments, the results showed that NR4A1 inhibited IL‐1β and NLRP3 promoter activity (Figure 3C and Figure S5C,D). Accordingly, the ChIP assay results showed that NR4A1 could bind to the promoter of IL‐1β and NLRP3 (Figure 3D and Figure S5E). These results importantly indicated NR4A1 transcriptionally inhibits NLRP3 and IL‐1β independent of NF‐κB.

**FIGURE 3:**
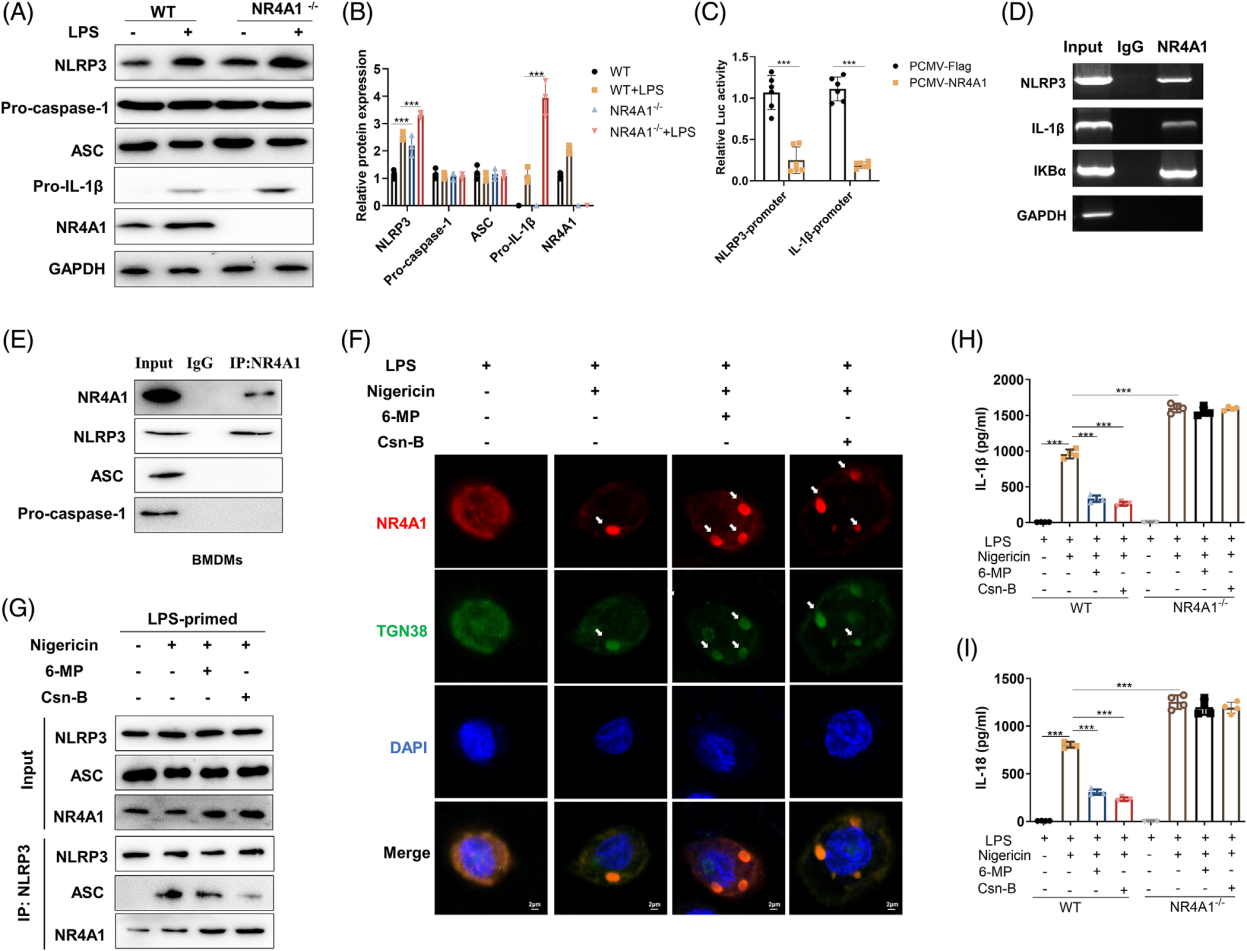
NR4A1 suppresses NLRP3 inflammasome activation by transcriptionally inhibiting NLRP3 and IL‐1β and co‐localizing with NLRP3 in trans‐Golgi. (A and B) Immunoblot analysis of NLRP3, pro‐caspase‐1, pro‐IL‐1β and ASC protein in WT and *NR4A1^‐/‐^* BMDMs. (C) The mice NLRP3 or IL‐1β promoter reporters, pTK and protein that contains mutations in its DNA‐binding domain‐flag or PCMW‐NR4A1 were transiently transfected into HEK293T cells; after 24 h, the dual‐luciferase activity was measured. (D) ChIP assay was used to measure the binding of NR4A1 on the NLRP3, IL‐1β, and IKBα (positive control) promoter in BMDMs. (E) IP and immunoblot analysis of the interaction of endogenous NR4A1 and NLRP3 inflammasome in BMDMs. (F) Immunofluorescence analysis NR4A1 and TGN38 in LPS‐primed BMDMs treated with 6‐MP (Mercaptopurine) and Csn‐B (Cyclosporine‐B) for 30 min and then stimulated with Nigericin for another 45 min. (G) IP and immunoblot analysis of the interaction of endogenous NLRP3 with NR4A1, ASC. (H and I) enzyme‐linked immunosorbent assay analyzed IL‐1β and IL‐18 in supernatants of WT and *NR4A1^‐/‐^* BMDMs. Values are expressed as mean ± SD, ^*^ *p *< 0.05, three independent experiments

Increasing evidence supports that NR4A1 function is also controlled at the post‐transcriptional level, predominantly via interaction, modification and subcellular localization.^7^, ^8^ We reconstructed NLRP3 inflammasome in HEK293T cells to eliminate transcriptional regulation, and overexpression of NR4A1 significantly decreases Nigericin‐induced NLRP3 inflammasome activation (Figure S6). Co‐immunoprecipitation showed that NR4A1 interacts with NLRP3 in macrophages (Figure 3E and Figure S7A). The association between NR4A1 and NLRP3 is substantially increased by LPS, Nigericin or ATP (Figure S7B). Through transfecting GFP‐NR4A1 and Flag‐NLRP3 in HEK293T cells, we found the interaction between NR4A1 and NLRP3 is enhanced by Nigericin or ATP, independently of the first signal (Figure S7C). To clarify the key domain of the interaction, the results showed that NR4A1 interacts with the NACHT domain of NLRP3, and NLRP3 interacts with the LBD domain of NR4A1 (Figure S7D‐F). For NLRP3‐ASC complex formation, after the LBD structure is deleted, the inhibitory effect of NR4A1 on NLRP3 specks is disappeared (Figure S7G). These results suggested that the LBD domain is the key domain for NR4A1 to inhibit NLRP3 inflammasome.

Interestingly, under the stimulation of Nigericin or ATP, NR4A1 and NLRP3 aggregate into specks and co‐localized together (Figure S8A,C). Usually, once NLRP3 inflammasome activation, NLRP3 migrates from the endoplasmic reticulum to trans‐Golgi or mitochondria.^9^, ^10^ We labeled the trans‐Golgi with TGN38, and NR4A1 co‐localizes with trans‐Golgi, after activating NLRP3 inflammasome (Figure S8B). NR4A1 is activated by 6‐MP (Mercaptopurine) and Csn‐B (Cyclosporine‐B), which promotes NR4A1 aggregating into specks and co‐localize in the trans‐Golgi (Figure 3F and Figure S9A,B). Interestingly, 6‐MP or Csn‐B promotes the interaction between NLRP3 and NR4A1 and decreases the interaction between NLRP3 and ASC (Figure 3G). For NLRP3 inflammasome, 6‐MP or Csn‐B suppresses ASC oligomerization, caspase‐1cleavage, IL‐1β and IL‐18 production (Figure S9C‐I). These results indicated that the agonist of NR4A1 promotes the migration of NR4A1 to trans‐Golgi to suppress NLRP3 inflammasome activation. We also confirmed the effect of NR4A1 agonists in vivo, these results showed that 6‐MP or Csn‐B, inhibit *C. rodentium*‐induced colitis and NLRP3 inflammasome activation (Figure S10). However, 6‐MP or Csn‐B could not inhibit Nigericin‐induced NLRP3 inflammasome activation in *NR4A1^−/‐^* BMDM (Figure 3H,I).

In summary, NR4A1 exerts a protective effect against *C. rodentium*‐induced colitis through inhibiting NLRP3 inflammasome. Mechanistically, NR4A1 not only transcriptionally inhibits the expression of NLRP3 and IL‐1β, but also interacts with NLRP3 and jointly migrates to trans‐Golgi, blocking NLRP3 activation and assembly (Figure 4). Our findings offer a new idea for the treatment of IBD, which is, targeting transcriptional regulation and trans‐Golgi translocation of NR4A1.

**FIGURE 4:**
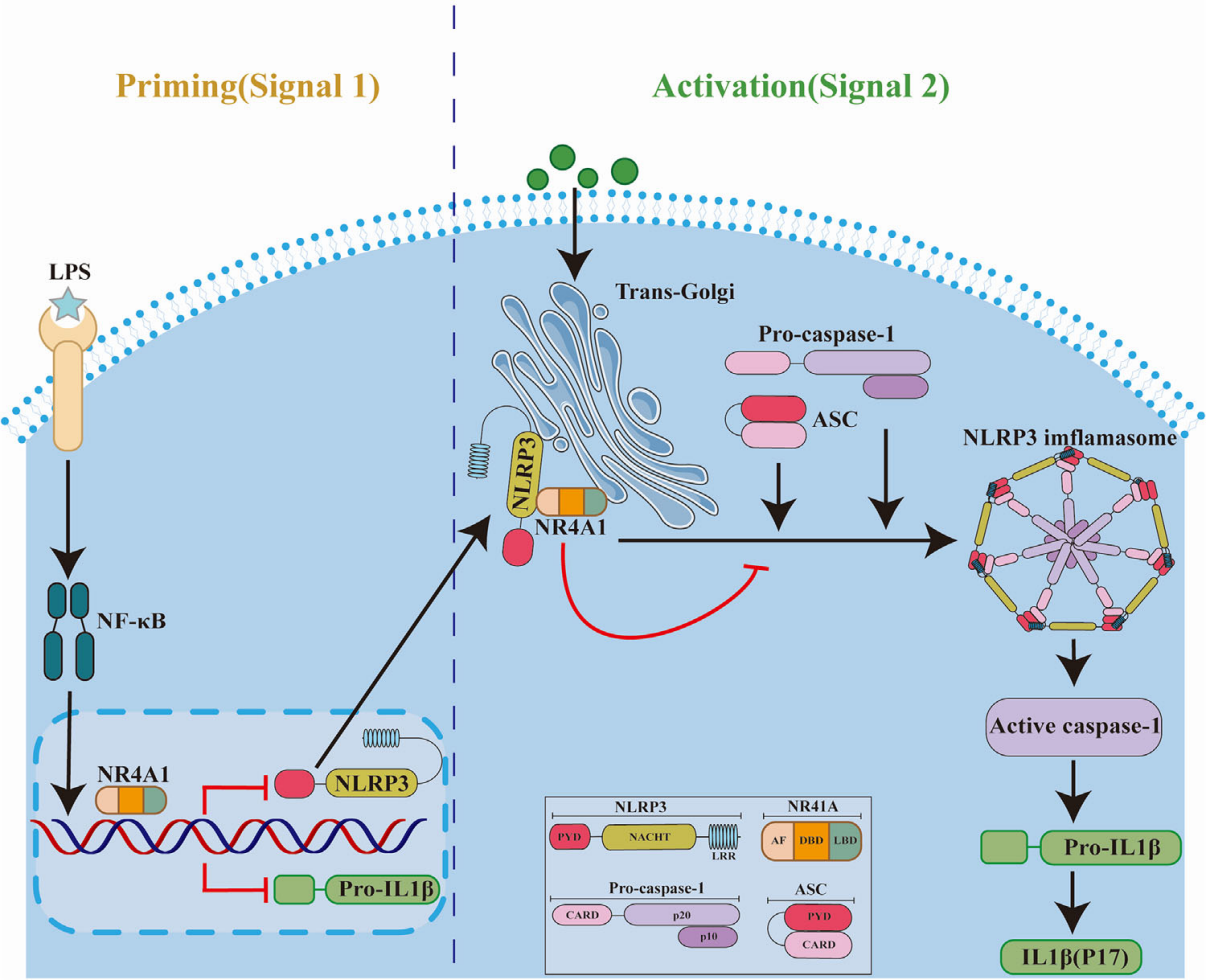
Putative mechanism for the mechanism of NLRP3 inflammasome activation regulated by Nu77 in macrophages. NR4A1 directly transcriptionally inhibits NLRP3 and IL‐1β expression; meanwhile, it directly interacts with NLRP3 and migrates to trans‐Golgi to inhibit NLRP3 assembly

## CONFLICT OF INTEREST

The authors declare that there is no conflict of interest that could be perceived as prejudicing theimpartiality of the research reported.

## Supporting information

Supporting information

Supporting information
