# Supplementary material for: NR4A1 suppresses pyroptosis by transcriptionally inhibiting NLRP3 and IL‐1β and co‐localizing with NLRP3 in trans‐Golgi to alleviate pathogenic bacteria‐induced colitis
Source: Clin Transl Med. 2021 Dec 19;11(12):e639. doi: 10.1002/ctm2.639 (PMC8684771; doi:10.1002/ctm2.639)
Supplement: Supplementary file 1 — Supporting information [file CTM2-11-e639-s001.docx]

**Supplementary materials**

NR4A1 suppresses pyroptosis by transcriptionally inhibiting NLRP3 and IL-1β and co-localizing with NLRP3 in trans-Golgi to alleviate pathogenic bacteria-induced colitis

Zhao Deng^1^, Zhipeng Yang^1^, Chenbin Cui^1^, Hong-Kui Wei^1^, Lijia Wang^3^, De'an Tian^3^, Fang Xiao^3^, Jian Peng^1,2*^

1 Department of Animal Nutrition and Feed Science, College of Animal Science and Technology, Huazhong Agricultural University, 430070, Wuhan, P. R. China
2 The Cooperative Innovation Center for Sustainable Pig Production, Wuhan 430070, Hubei, China

3 Department of Gastroenterology, Tongji Hospital of Tongji Medical College, Huazhong University of Science and Technology, Wuhan 430030, Hubei Province, China
*Corresponding author:

E-mail address:

[pengjian@mail.hzau.edu.cn](mailto:pengjian@mail.hzau.edu.cn)

**Acknowledgements**

We thank the Department of Animal Nutrition and Feed Science (Huazhong Agricultural University) for their help. This research was supported by the National Natural Science Foundation of China (31972578), Hubei province technology innovation special major project (2019ABA081), Basic scientific research operating expenses of central universities (2662017PY017).

**Conflict of interest**

The authors have declared that no conflicts of interest exist.

**Data Availability**

The data used to support the findings of this study are available from the corresponding author upon request.

**Author contributions**

Zhao Deng conducted almost experiments and wrote the manuscript. Zhipeng Yang completed the figure 8 and some experiment. Chenbin Cui completed some experiment. Hong-Kui Wei helped to design the experiment. Lijia Wang, De'an Tian, and Fang Xiao provided the help. Jian Peng designed the experiment and provided funding, and modified the manuscript.

**Materials and methods**

Plasmids, reagents, and antibodies

*Human Flag-NLRP3, Flag-LRR, Flag-PYD, and Flag-NACHT were gifts from* Professor Rongbin Zhou (University of Science and Technology of China). Human GFP-NLRP3, Myc-ASC, Flag-caspase-1, and Flag-IL-1β *were gifts from* Professor Anding Zhang (Huazhong Agricultural University). Human Flag-NR4A1, GFP-NR4A1, RFP-NR4A1 were PCR-amplified from cDNA constructs (2×PrimeSTAR GC Buffe, TaKaRa, Beijing, China) and cloned into the expression vector. Mouse Myc-NR4A1, Myc-NR4A1-AF, Myc-NR4A1-DBD, Myc-NR4A1-LBD, Flag-NLRP3 were PCR-amplified from cDNA constructs and cloned into the expression vector. Mouse Pgl3-NLRP3-promoter-2000bp, Pgl3-NLRP3-deletion-promoter-2000bp, Pgl3-NLRP3-mutation-promoter-2000bp, Pgl3-IL-1β-promoter-2000bp, Pgl3-IL-1β-mutation-promoter-2000bp, Pgl3-IL-1β-deletion-promoter-2000bp were PCR-amplified from cDNA constructs and cloned into the Pgl3-basic vector.

*C. rodentium* is a gift from Professor Shan Li (Huazhong Agricultural University). LPS (L6230) was purchase from Sigma-Aldrich (St. Louis, MO, USA). Protease Inhibitor Cocktail (C0001), Nigericin (T3092), and 6-MP (T2201) were purchased from Target Mol (Shanghai, China). Csn-B (HY-N2148 T3976) was purchased from MedChemExpress (Shanghai, China). ATP (A600020) was purchased from Sangon Biotech (Shanghai, China). Cell lysis buffer for Western analysis (P0013), phenylmethanesulfonyl fluoride (PMSF) (ST505), and DAPI (P0131) were purchased from Beyotime (Shanghai, China). Human Interleukin 6 (IL-6) ELISA kit (2H-KMLJ31201mm), IL-1β ELISA Kit (2H-KMLJh310351), IL-22 ELISA Kit (2H-KMLJh310356), TNF-α ELISA Kit (2H-KMLJh311776) were purchased from Nanjing Camilo biological  engineering  co.LTD (Nanjing, China).

The antibodies against pro-IL-1β (A16288), GSDMD (A20197), β-actin (AC026), β-tubulin (AC026) were purchased from Abclonal (Wuhan, China). The antibody against mouse IL-1β (63124) was obtained from Cell Signaling Technology (Shanghai, China). The ChIP antibody against NR4A1 (ab13851) was purchased from Abcam (Shanghai, China). The antibody against NR4A1 (DF7850) was purchased from Affinity (Cincinnati, USA). The antibody against NLRP3 (AG-20B-0014-C100), and mouse caspase-1 (AG-20B-0042) were brought from Adipogen (San Diego CA, USA). The antibody against ASC (sc-514414) was brought from Santa Cruz Biotechnology (Dallas, USA). The antibodies against GAPDH (D190090), GFP (D110008) were purchased from Sangon Biotech (Shanghai, China). The antibodies against Flag (20543-1), Myc (16286-1-AP), TGN38 (66477-1) were purchased from Proteintech (Whan, China).

Cell culture, stimulation, and transfection

Bone marrow macrophages were derived from C57BL/6 mice and cultured in DMEM complemented with fetal bovine serum (FBS, Gibco, San Diego, CA, USA) and 1% penicillin/streptomycin at 37°C under a 5% CO_2_ atmosphere., supplemented with 20% L929 cell supernatant.

Human acute monocytic leukemia THP-1 cells were obtained from BeNa Culture Collection (Beijing, China). THP-1 cells were cultured in RIPA 1640 containing 10% fetal bovine serum (FBS, Gibco, San Diego, CA, USA) and 1% penicillin/streptomycin at 37°C under a 5% CO_2_ atmosphere. Differentiation of THP-1 cells were induced by 100nM phorbol 12-myristate 13-acetate (PMA) for 6 h.

HEK293T cells were cultured in Dulbecco’s modified Eagle’s medium (DMEM), containing 10% fetal bovine serum and 1% penicillin/streptomycin at 37°C under a 5% CO_2_ atmosphere.

For induction of canonical NLRP3 inflammasome activation, 5 × 10^5^ macrophages were plated overnight in 12-well plates and the medium was changed to Opti-MEM (1% FBS) in the following morning, and then cells were treated with 200 ng/ml LPS for 4 h. After that, the cells were stimulated for 4 h with MSU (200µg/ml), or for 30 min with ATP (5 mM) or Nigericin (10 μM).

For AIM2 inflammasome activation, poly(dA:dT) (1 μg/ml) was transfected into BMDMs for 4 h using Lipofectamine 2000. For NLRC4 inflammasome activation, Salmonella was grown in Luria-Bertani (LB) for 4 h, and BMDMs were infected for 1 h with the salmonella culture (1:100) and then incubated for another 3 h in the presence of gentamycin.

For induction of non-canonical NLRP3 inflammasome activation, 5 × 10^5^ macrophages were plated overnight in 12-well plates and the medium was changed to Opti-MEM (1% FBS) in the following morning, and then cells were treated with 400 ng/ml Pam3CSK4 (InvivoGen) for 4 h. After that, cells were transfected with cytoplasm LPS (cLPS) (500ng/ml) for16 h.

Transient transfection of HEK293T cells was transfected with Lipofectamine 2000 (Invitrogen, Carlsbad, CA, USA). The THP-1 cell lines that stably expressed *NR4A1*-specific shRNA (sh*-NR4A1*) and scrambled shRNA control (shCtrl) were constructed using a lentivirus vector-based shRNA technique. The human *NR4A1* shRNA target was 5′-TACACAGGAGAGTTTGACA-3′. Oligonucleotides were constructed in GV248 lentiviral RNAi vector (GeneChem, Shanghai, China) ^20^.

Animals

C57BL/6 mice (six weeks old) obtained from the Animal Experiment Center at Huazhong Agricultural University (Wuhan, China) were used for the present study. *NR4A1^-/-^* (*Nur77^-/-^*) mice were obtained from Cyagen (Guangzhou, China) ^19^. The mice were housed under specific pathogen-free conditions in an airconditioned room at 23±2^°^C. Food and water were supplied ad libitum. Animal welfare and experimental procedures were carried out following the criteria outlined in the Guide FOR THE CARE AND USE OF LABORATORY ANIMALS (Eight Edition) and the related ethical regulations of Huazhong Agricultural University. All animal experimental protocols were approved by the Institutional Animal Care and Use Committee of Huazhong Agricultural University. The ethical approval number is HZAUMO-2020-0055. All efforts were made to minimize animal suffering and to reduce the number of animals used.

Establishment of *C. rodentium*-induced mice colitis model

Male C57BL/6 mice (six weeks old) were treated with 5×10^9^ CFU *C. rodentium* via oral gavage for once. After 7 days, the mice were humanely euthanized. Serum and colon samples were collected, and sectioned for further analysis.

Subsequently, to further confirm the effects of 6-MP and Csn-B, 6-MP or Csn-B were injected intraperitoneally with 10mg/kg daily from the second day. At day 7, the mice were humanely euthanized.

In vivo LPS challenge

Female C57BL/6 mice (six weeks old) control mice were injected intraperitoneally with LPS (10 mg/kg). The serum samples were collected after 6 h to detect serum cytokine levels, and the colons were excised, measured, and sectioned for further analysis.

Intestine histological assessments

For H&E staining, tissues were fixed in 10% neutral-buffered formalin and embedded in paraffin. Sections of tissue 5 μm in thickness were affixed to slides and then stained with haematoxylin and eosin (H&E), according to standard histological procedures.

Cell Death assay

Cell death was measured by LDH release assay (Promega, Beijing, China), following the manufacturer’s instructions. The culture media were collected and centrifuged at 12000g for 5 min to remove cell debris. LDH release in the media was measured at OD 490. The relative LDH release was expressed as the percentage LDH activity in supernatants of cultured cells (medium) compared with total LDH (from media and the cells) and used as an index of cytotoxicity.

Reconstitution of NLRP3 Inflammasome in HEK293T cells

The HEK293T cells were seeded into 6-well plates at 5×10^5^ per well in complete cell culture medium. After 12 h, cells were transfected with plasmids expressing Flag-IL-1β (0.5 μg), Flag-caspase-1 (0.1 μg), Myc-ASC (0.1 μg), GFP-NLRP3 (0.3 μg), and Flag-NR4A1 (1 μg). 36 h later, HEK293T cells treated with Nigericin (10 μm) for 30 min, cell supernatants were collected and analyzed for the IL-1β and caspase-1 maturation, and NLRP3-Speck was measured by immunofluorescence.

Luciferase reporter assays

The luciferase vector, expression vector, and renilla luciferase-expressing plasmid (pTK) were transfected into HEK293T cells for 6 h. After 24 h, washing twice with PBS, the cells were lysed using Dual-Glo luciferase reagent (YPH, Beijing, China). The luciferase activity was determined using a dual-luciferase reporter assay system and luminometer (Dynex Technologies, UK). The luciferase values were normalized to the Renilla values. The transfection experiments were performed in triplicate for each independent experiment.

ASC oligomerization assay

For ASC-speck formation, BMDMs were seeded at 2.5×10^6^ /ml on chamber slides and allowed to attach overnight. The following day cells were primed with LPS and treated with Nigericin. Cells were fixed with 4% paraformaldehyde followed by ASC and DAPI staining.

Immunoblot analysis and immunoprecipitation (IP) assay

The cells or tissues were extracted with protein lysis buffer (Beyotime, China) supplemented with protease inhibitor cocktail. The protein concentration was determined using the BCA Kit (Beyotime, China). Proteins (25–35 μg) were separated on a 10% polyacrylamide precast SDS gel (Bio-Rad, Richmond, CA, USA) followed by blotting on PVDF membranes (Millipore Billerica, MA, USA). The membrane was blocked with Tris-buffered saline (TBS) TBS and 0.1% (v/v) Tween-20 (TTBS) buffer (10 mM Tris-HCl (pH 7.6), 150 mM NaCl, 0.1% Tween) con- taining 5% skim milk powder for 2 h, and then incubated with the primary antibody overnight at 4℃. Secondary antibodies, anti-mouse, or rabbit IgG-HRP were used to detect primary antibodies. Binding was detected using an enhanced chemiluminescence detection kit (Thermo Fisher Scien- tific, San Jose, CA, USA) according to the manufacturer's instructions. Densitometry was performed using Image-J software (National Institutes of Health, Bethesda, MD, USA).

For IP experiments, the cells were lysed in IP buffer (Beyotime, China) and incubated with IP-grade antibodies, followed by pull-down with protein A/G beads (161-4023) (Bio-Rad, Richmond, CA, USA) for subsequent immunoblot analyses.

Immunofluorescence and confocal imaging

Cells were washed twice with PBS and fixed in 4% paraformaldehyde for 30 min at room temperature. 4,6-diamidino-2-phenylindole (DAPI) was used to label DNA. Confocal imaging was performed using a confocal laser scanning microscope (Carl Zeiss, Germany) equipped with an incubation chamber and a motorized table. Mitochondria were marked by MitoTracker (Red) (1:10 000 dilution) for 30 min before being fixed by 4% buffered formalin/PBS.

ELISA

Culture supernatants from cell culture, tissue culture and serum were collected, and levels of IL-1β and IL-18 (NEOBIOSCIENCE, Shenzhen, China) were determined according to manufacturer’s instructions.

Statistical analysis

No samples or animals were excluded from the analysis. Data were presented as the mean ± standard deviation. All data were accorded with normal distribution, and no variation was present. Differences between group means were determined by one-way ANOVA using SAS 8.0 software. The Tukey post hoc multiple comparison test was performed to compare significant variations. Differences were considered as significant at *P* < 0.05.

**Table S1**. **Potential monomeric NR4A1 binding sites (NBREs) in the promoter of IL-1β and NLRP3**

| **Consensus sequence** | **AAAGGTCA or TGACCTTT** |
| --- | --- |
| Potential NBREs in  Mouse IL-1β promoter | -1556 AGAACTGACCATTGCTCA -1538 |
| Potential NBREs in  Mouse NLRP3 promoter | -698 GTCTCTGCCCTTTCACCT -680 |

**Table S2**. **Primers used for RT-PCR analysis**

| Primer | Primer sequences (5′ → 3′) | Product size (bp) |
| --- | --- | --- |
| m-β-actin | GGCACCACACCTTCTACAATG  GGGGTGTTGAAGGTCTCAAAC | 133 |
| m-GAPDH | aactttggcattgtggaagg  ggatgcagggatgatgttct | 132 |
| m-NR4A1 | ATGCTTCGTGTCAGCACTAT  GTACTTGGCGCTTTTCTGTA | 86 |
| m-chip-NLRP3 | GCTCCTGTCTTCCTGAGTCTG TGTAGAACCACTGTCTGACCAT | 204 |
| m-chip-IL-1β | ACAGGACCTCTGGATTGTCTG CGATTTCAGGAAGGCAAACAGA | 178 |
| m-chip-IKBα | ATATGTCCGTCGTCGCAGAA  AGCTTCCAAGGACGCAGAC | 157 |
