# Supplementary material for: NR4A1 suppresses pyroptosis by transcriptionally inhibiting NLRP3 and IL‐1β and co‐localizing with NLRP3 in trans‐Golgi to alleviate pathogenic bacteria‐induced colitis
Source: Clin Transl Med. 2021 Dec 19;11(12):e639. doi: 10.1002/ctm2.639 (PMC8684771; doi:10.1002/ctm2.639)
Supplement: Supplementary file 2 — Supporting information [file CTM2-11-e639-s002.pdf]

Fig. S1

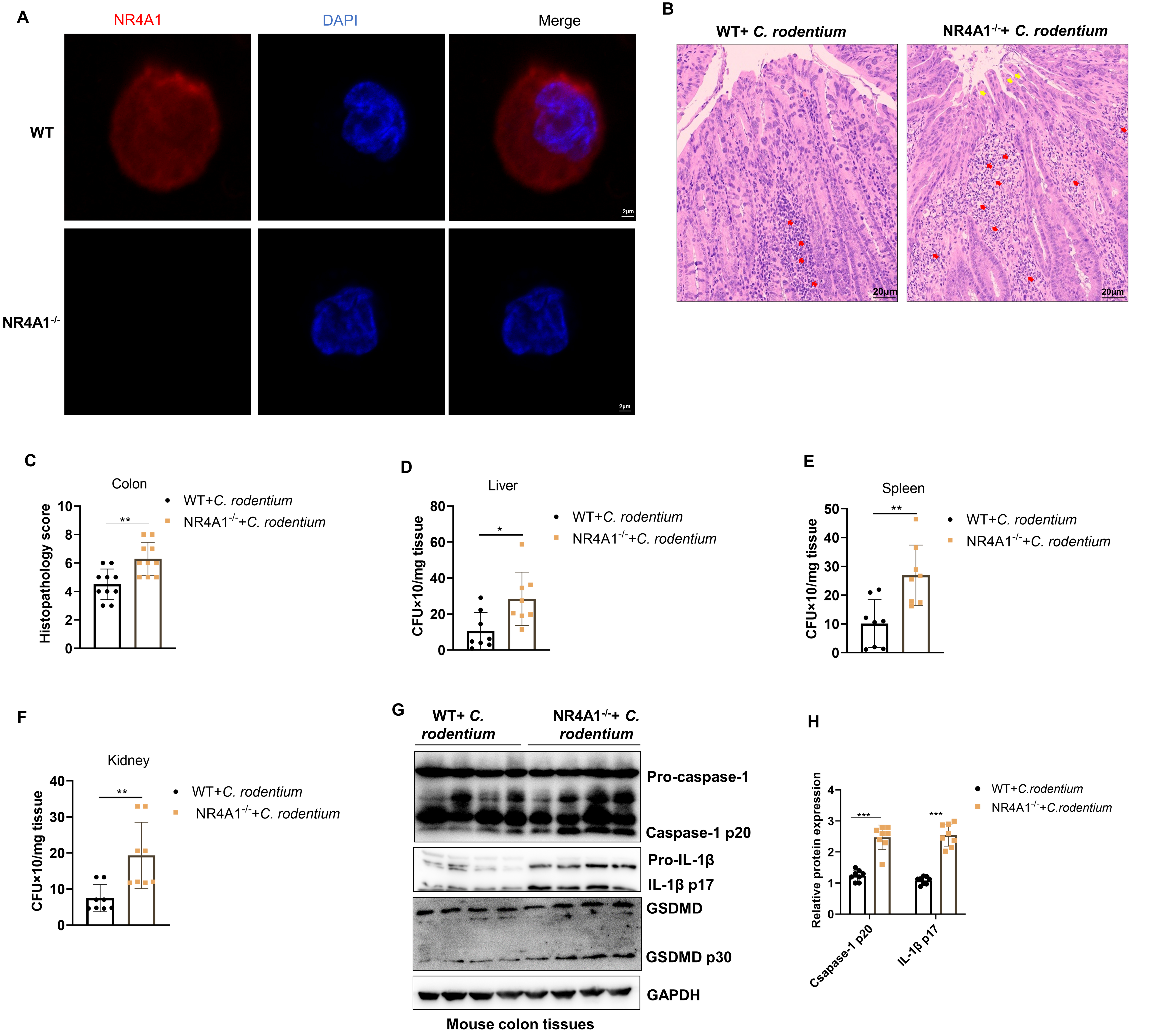

Figure S1. NR4A1 deficiency exacerbates mice colitis .

(A) Immunofluorescence analysis of NR4A1 in WT and *NR4A1*$^{-/-}$ BMDMs (n = 3/group). (B, C) Images and histopathological scores of the colon tissues by H&E statining. (D-F) The number of *C. rodentium* colonized in the liver, spleen, and kidney (n = 10/group) . (G, H) Immunoblot analysis of caspase-1, IL-1β and GSDMD protein in mice colon tissue (n = 6/group) . Values are expressed as mean ± SD, *P < 0.05.

Fig. S2

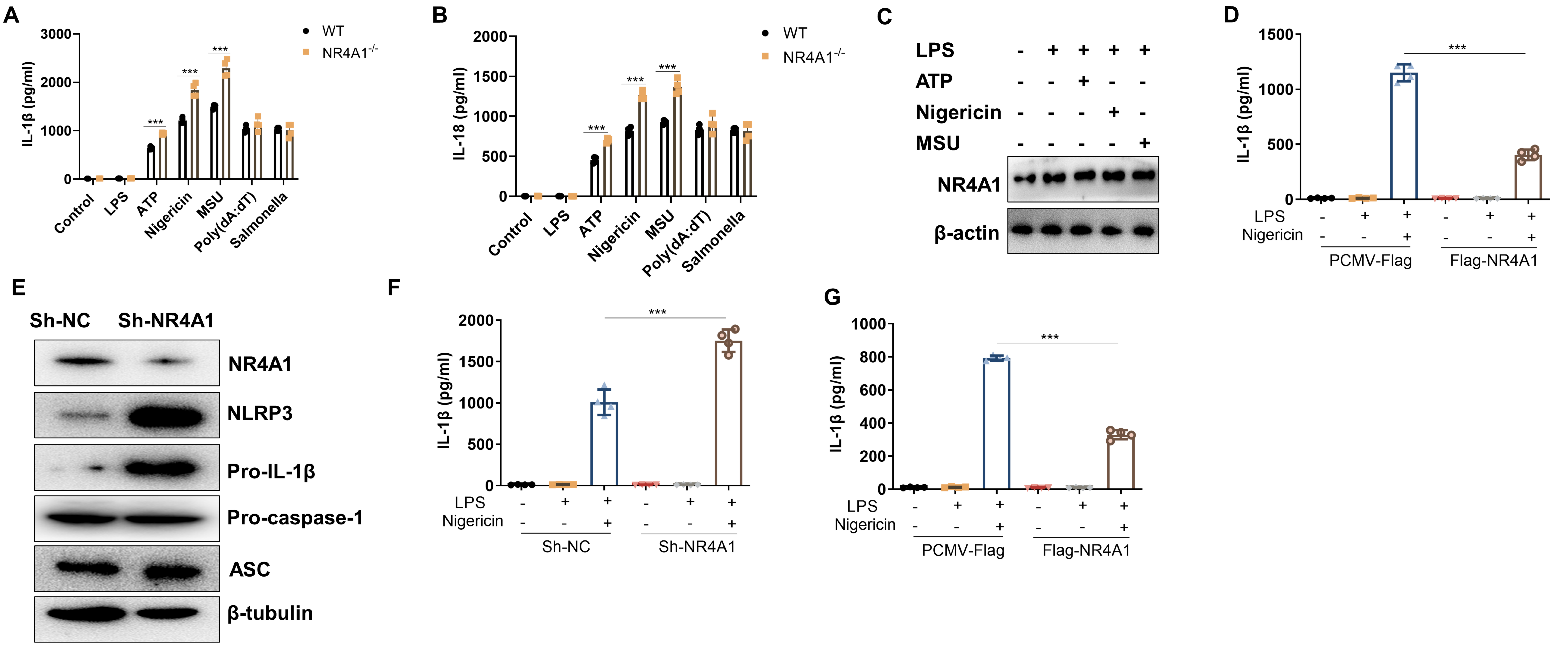

Figure S2. NR4A1 inhibits canonical NLRP3 inflammasome activation in macrophages.

(A, B) ELISA analyzed IL-1β and IL-18 in supernatants of LPS-primed BMDMs treated with MSU, Nigericin, ATP, poly (dA:dT), salmonella in WT and *$NR4A1^{-/-}$* BMDMs. (C) BMDMs were primed for 4 h with LPS (200 ng/mL), then stimulated with Nigericin (10 μM) for 45 min, immunoblot analysis of NR4A1 in cell lysates. (D) BMDMs were transfected with PCMV-Flag, Flag-NR4A1 for 72 h, then primed for 4 h with LPS (200 ng/mL), then stimulated with Nigericin (10 μM) for 45 min, ELISA analyzed IL-1β in supernatants.

Differentiation of THP-1 cells were induced by 100nM PMA for 4 h, NR4A1 was knockdown by sh-NR4A1 for 12 h. After 60 h, THP-1 cells were primed with LPS (200 ng/mL) for 4 h, followed by stimulation with Nigericin (10 μm) for 45 minutes. (E) Immunoblot analysis of NR4A1, NLRP3, pro-IL-1β, pro-caspase-1 and ASC in cell lysates. (F) ELISA analyzed IL-1β in supernatants. (G) THP-1 cells were transfected with PCMV-Flag, Flag-NR4A1 for 72 h, then primed for 4 h with LPS (200 ng/mL), then stimulated with Nigericin (10 μM) for 45 min, ELISA analyzed IL-1β in supernatants. Values are expressed as mean ± SD, *$P < 0.05$, three independent experiments.

Fig. S3

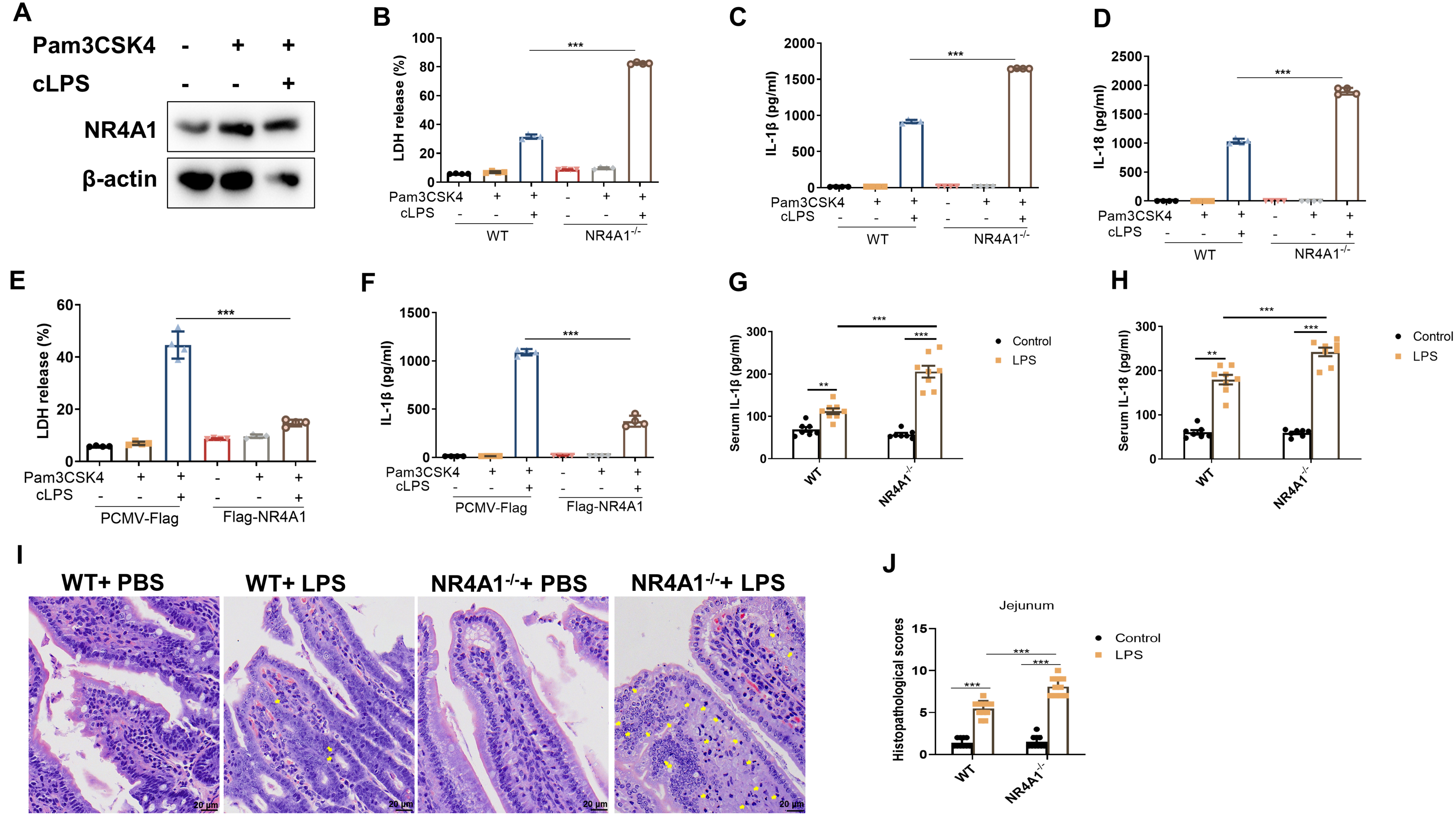

Figure S3. NR4A1 inhibits non-canonical NLRP3 inflammasome activation.

BMDMs were primed with Pam3CSK4 for 4 h, followed by cLPS treatment for 16 h. (A) Immunoblot analysis of NR4A1 in cell lysates. (B-D) Supernatants were analyzed for LDH, IL-1β, and IL-18. (E, F) BMDMs were transfected with PCMV-Flag, Flag-NR4A1 for 72 h, then were primed with Pam3CSK4 for 4 h, followed by cLPS treatment for 16 h, supernatants were analyzed for LDH and IL-1β. Three independent experiments.

WT and *NR4A1*$^{-/-}$ mice were intraperitoneal injected with PBS or LPS (10mg/kg) for once, mice were sacrificed after 6 h (n=10/group). (G, H) Production of IL-1β and IL-18 in mice serum. (I, J) H&E stains of serial sections of jejunum, histopathological scores of each group were determined. Values are expressed as mean ± SD, *P < 0.05.

Fig. S4

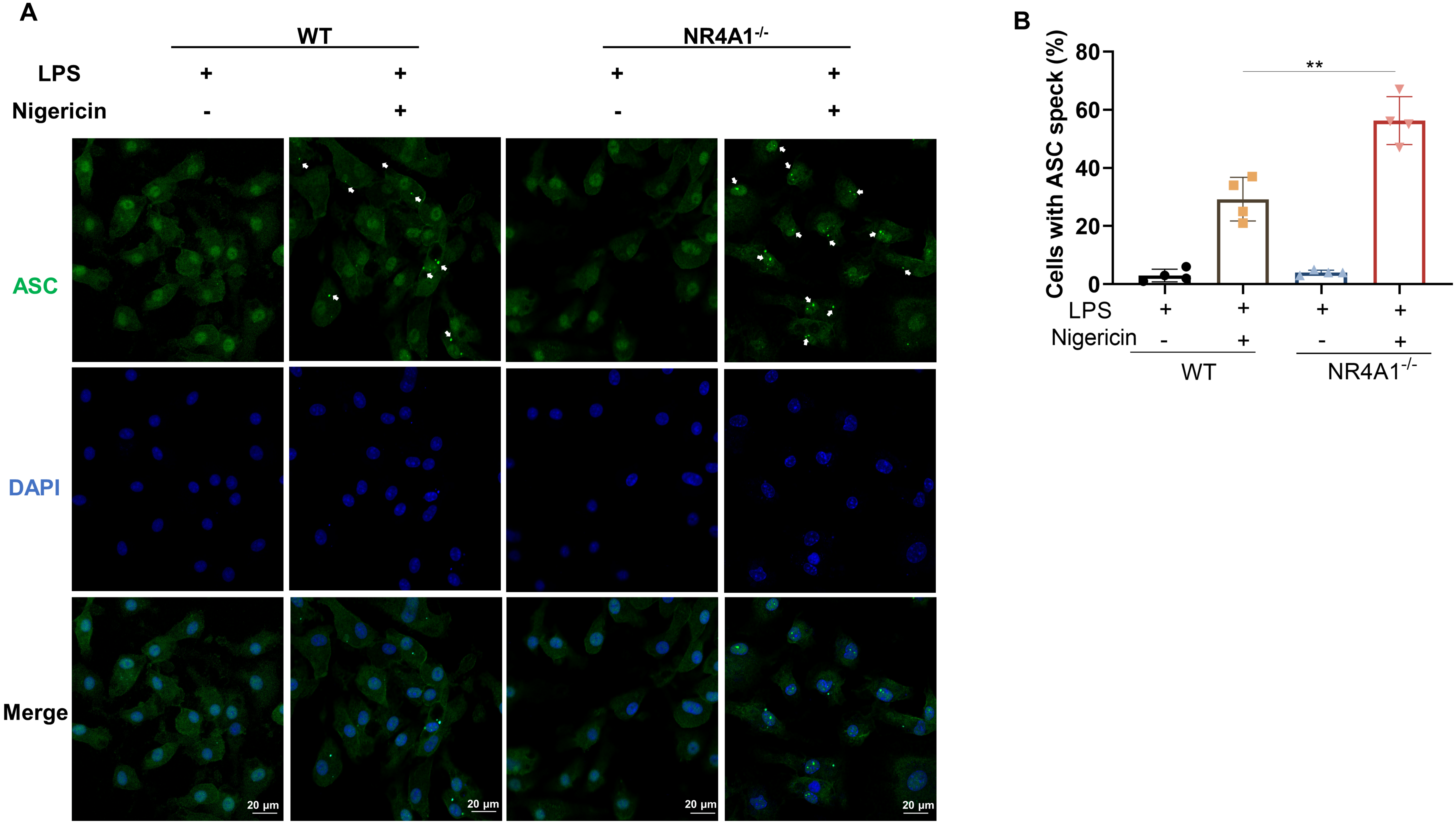

Figure S4. NR4A1 inhibits NLRP3 inflammasome assembly.

(A, B) Immunofluorescence analysis and quantification of ASC specks. Values are expressed as mean ± SD, *P < 0.05, three independent experiments.

Fig. S5

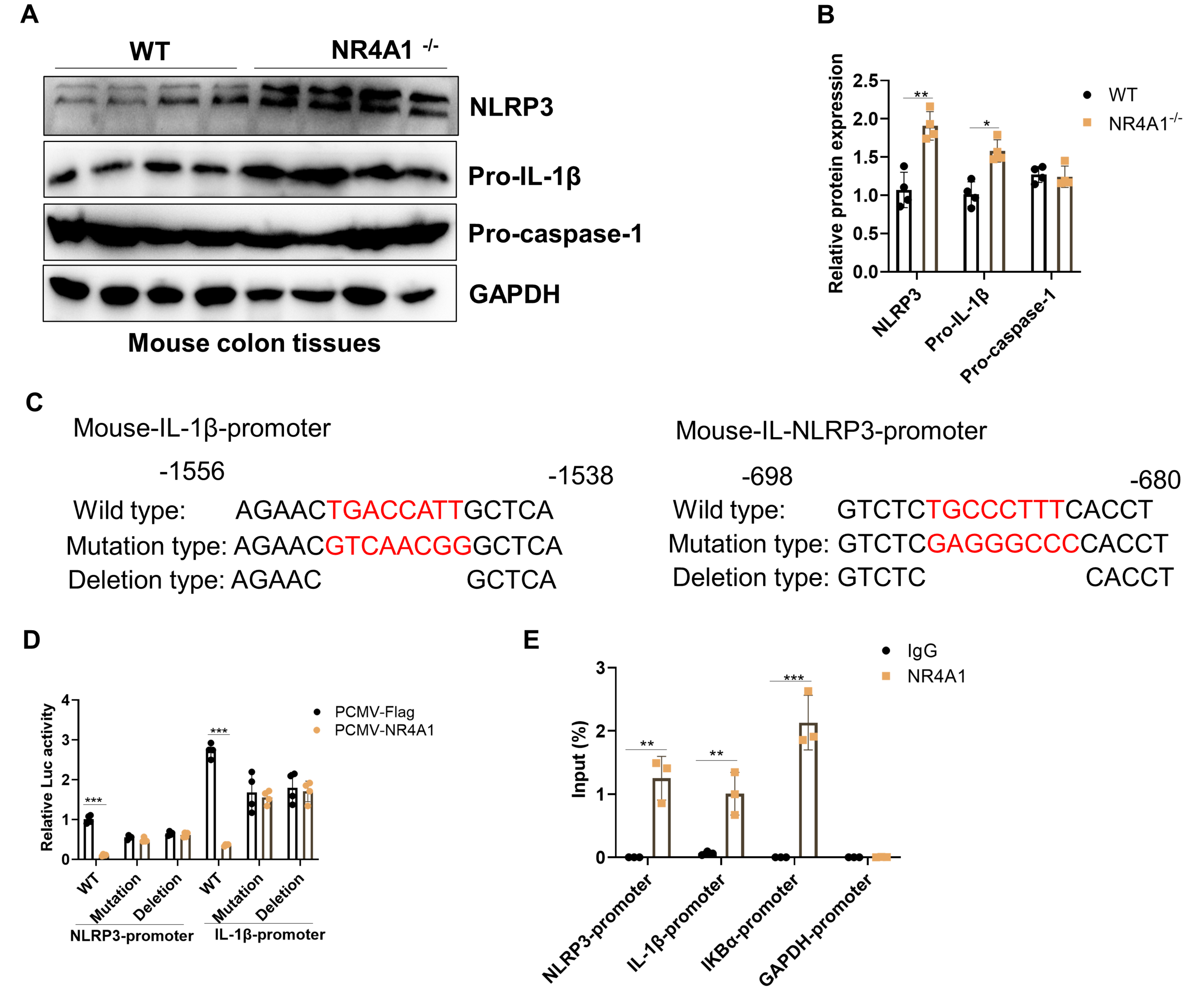

Figure S5. NR4A1 transcriptionally inhibit NLRP3 and IL-1β.

(A, B) Immunoblot analysis of NLRP3, pro-caspase-1, and pro-IL-1β protein in colon tissues of WT and *NR4A1*$^{-/-}$ mice (n = 6/group).

(C, D) Wild-type (WT) promoter, mutation or deletion of mice NLRP3 or IL-1β promoter reporters, pTK, and PCMV-flag or PCMV-NR4A1 were transiently transfected into HEK293T cells, after 24 h the dual-luciferase activity was measured. (E) ChIP-qPCR assay were used to measure the binding of NR4A1 on the NLRP3, IL-1β, IKBα (positive control), and GAPDH promoter in BMDMs. Values are expressed as mean ± SD, *P < 0.05, three independent experiments.

Fig. S6

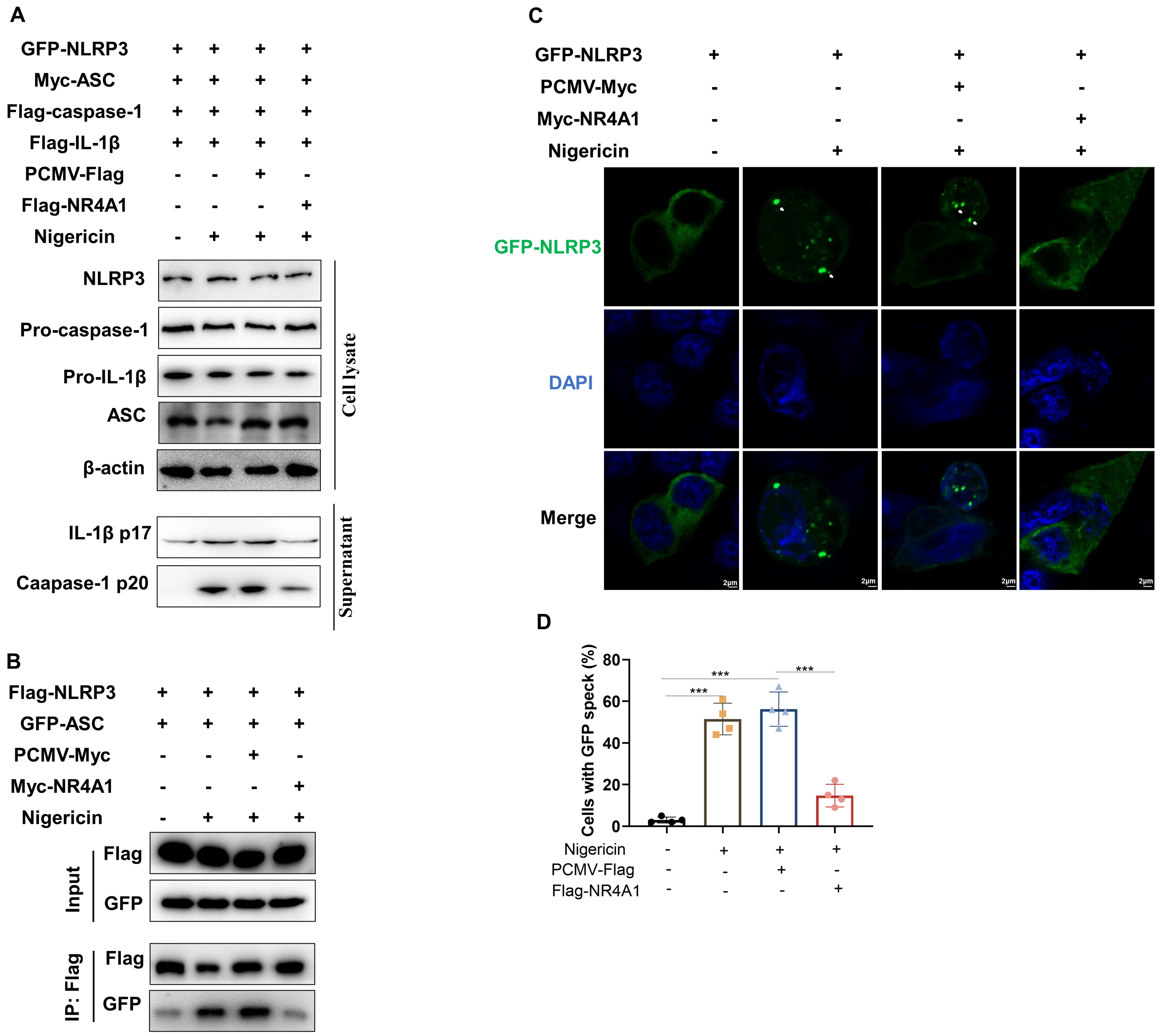

Figure S6. NR4A1 inhibits NLRP3 inflammasome in a non-transcribed manner.

The HEK293T cells were transfected with plasmids expressing Flag-IL-1β, Flag-caspase-1, Myc-ASC, GFP-NLRP3, and Flag-NR4A1, after 36 h, HEK293T cells treated with Nigericin (10 μm) for 45 min. (A) Immunoblot analysis of IL-1β p17 and caspase-1 p20 in supernatants, and immunoblot analysis of NLRP3, pro-caspase-1, and pro-IL-1β in cell lysates.

The HEK293T cells were transfected with plasmids expressing Flag-IL-1β, Flag-caspase-1, Myc-ASC, GFP-NLRP3, and Flag-NR4A1, after 36 h, HEK293T cells treated with Nigericin (10 μm) for 45 min. (B) IP and immunoblot analysis of the interaction of exogenous NLRP3 and ASC. (C, D) Immunofluorescence analysis and quantification of GFP-NLRP3 specks in HEK293T cells. Values are expressed as mean ± SD, *$P < 0.05$, three independent experiments.

Fig. S7

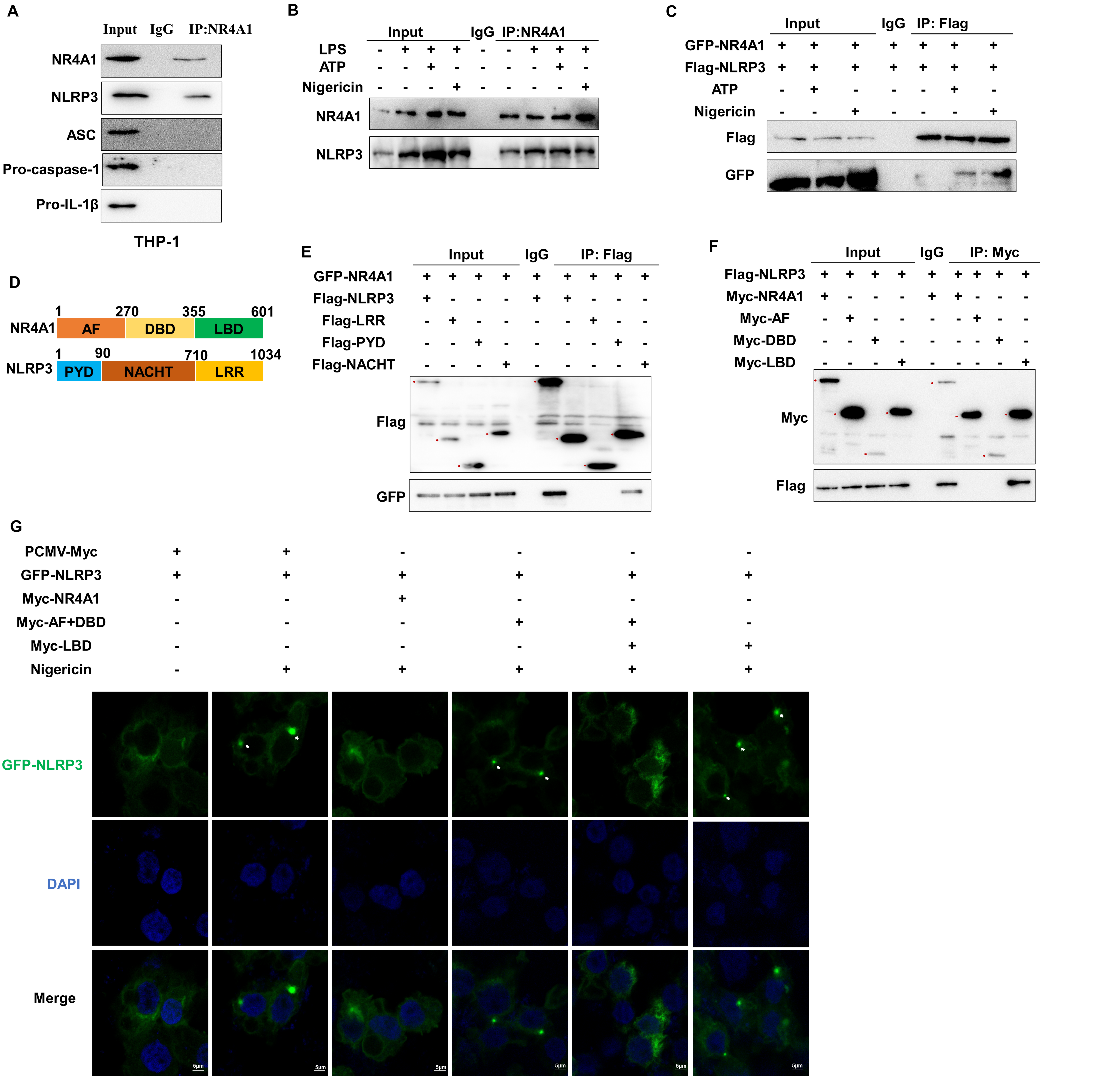

Figure S7. NR4A1 directly interacts with NLRP3 to suppress its activation

(A) IP and immunoblot analysis of the interaction of endogenous NR4A1 and NLRP3 inflammasome in THP-1 cells. (B) IP and immunoblot analysis of the interaction of endogenous NR4A1 and NLRP3 in LPS-primed BMDMs treated with ATP or Nigericin for 45 min. (C) IP and immunoblot analysis of the interaction of exogenous GFP-NR4A1 and Flag NLRP3 in HEK293T cells treated with ATP or Nigericin for 45 min. (D) Domains of NR4A1 and NLRP3. (E) HEK293T cells were transfected with GFP-NR4A1 and Flag-NLRP3, or Flag-LRR, Flag-PYD, or Flag-NACHT, IP and immunoblot analysis of GFP- and Flag-tagged proteins in cell lysates immunoprecipitated with anti-Flag ImpetiCbead. (F) HEK293T cells were transfected with Flag-NLRP3 and Myc-NR4A1, or Myc-AF, or Myc-DBD, or Myc-LBD, IP and immunoblot analysis of Flag- and Myc-tagged proteins in cell lysates immunoprecipitated with anti- Myc ImpetiCbead. (G) Immunofluorescence analysis of GFP-NLRP3 specks in HEK293T cells, three independent experiments.

Fig. S8

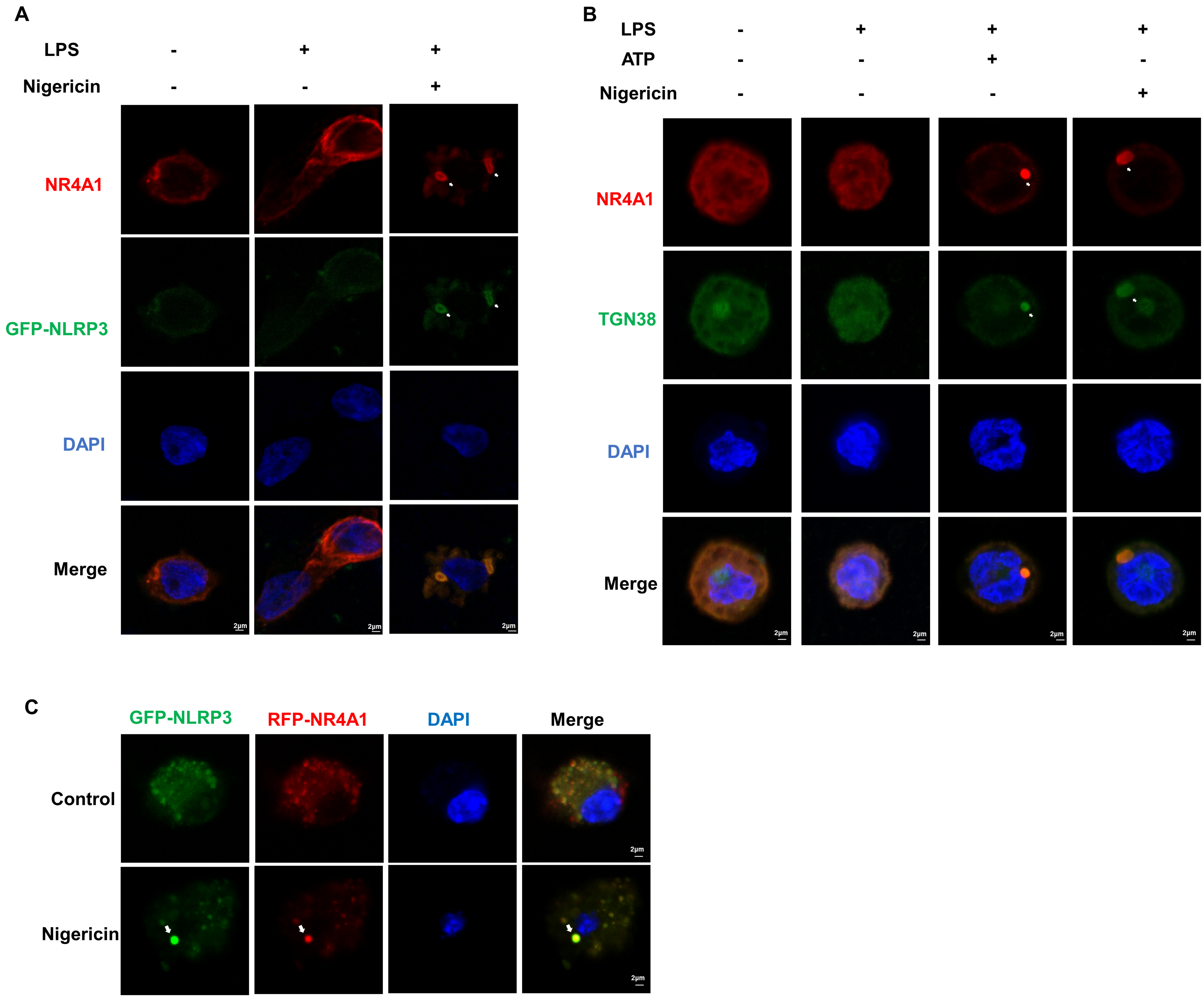

Figure S8. NR4A1 interacts with NLRP3 and co-localizes in the trans-Golgi.

BMDMs were transfected with GFP-NLRP3 for 72 h, then treated with LPS (200 ng/mL) for 4 h, followed by stimulation with Nigericin (10 μM) for 45 min. (A) Immunofluorescence analysis NR4A1 and GFP-NLRP3. (B) Immunofluorescence analysis NR4A1 and TGN38 in LPS-primed BMDMs treated with ATP or Nigericin for 45 min. (C) THP-1 cells were transfected with GFP-NLRP3 and RFP-NR4A1 for 72 h, then treated with Nigericin (10 μM) for 45 min, Immunofluorescence analysis RFP-NR4A1 and GFP-NLRP3 .Three independent experiments.

Fig. S9

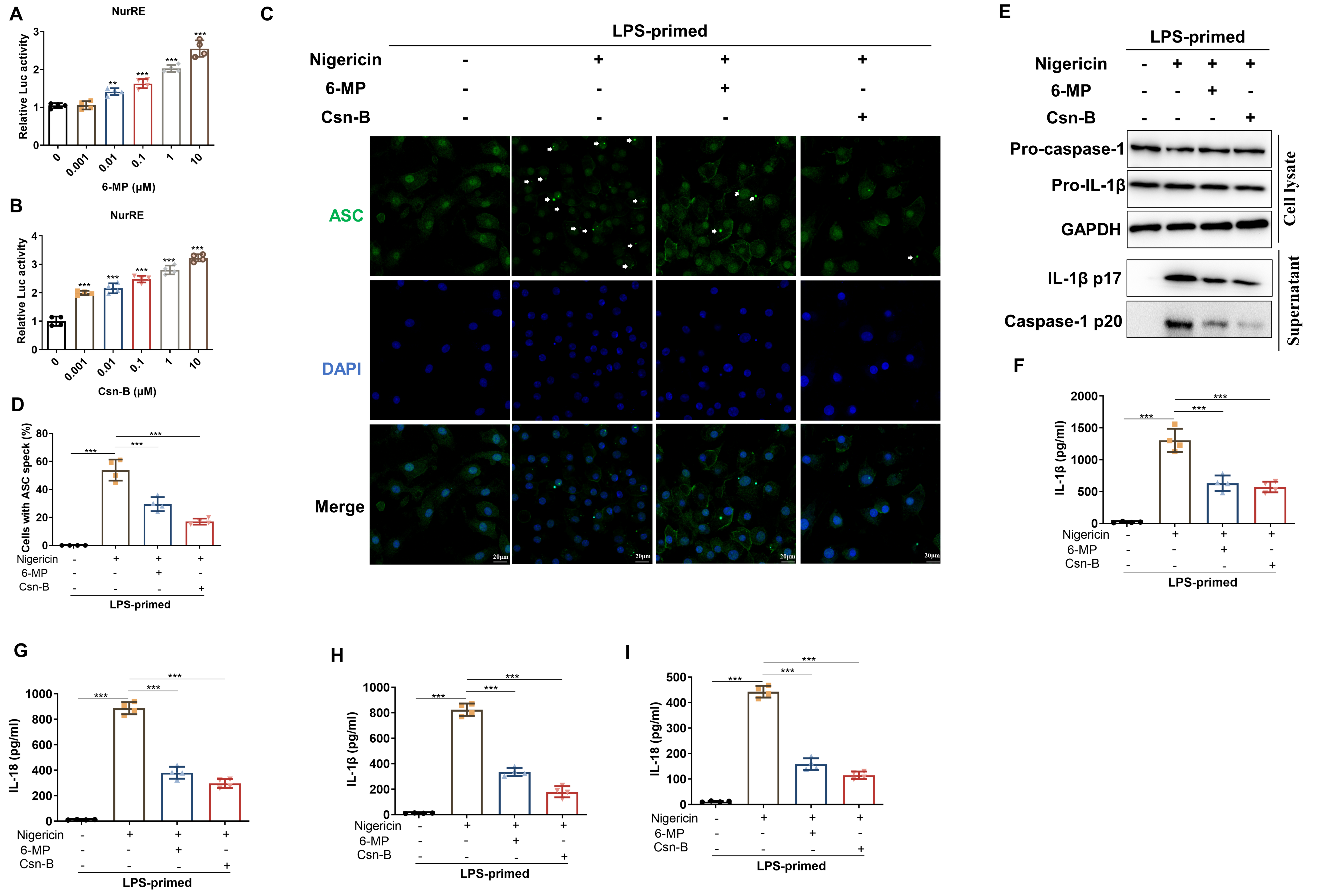

Figure S9. 6-MP and Csn-B inhibits NLRP3 inflammasome activation.

(A, B) Reporter genes for NR4A1 (NurRE) together with renilla luciferase-expressing plasmid (pTK) were transiently transfected into HEK293T cells. After 16 h of transfection, the cells were treated with 6-MP or Csn-B for 12 h at different concentrations as indicated, and the activities of the reporter gene were determined by luciferase assay and normalized to the pTK activity. (C, D) Immunofluorescence analysis and quantification of ASC specks in BMDMs. (E) Immunoblot analysis of IL-1β p17 and caspase-1 p20 in supernatants, and immunoblot analysis of pro-caspase-1, and pro-IL-1β in cell lysates of BMDMs. (F, G) ELISA analyzed IL-1β and IL-18 in supernatants of BMDMs. (H, I) ELISA analyzed IL-1β and IL-18 in supernatants of THP-1 cells. Values are expressed as mean ± SD, *$P < 0.05$, three independent experiments.

Fig. S10

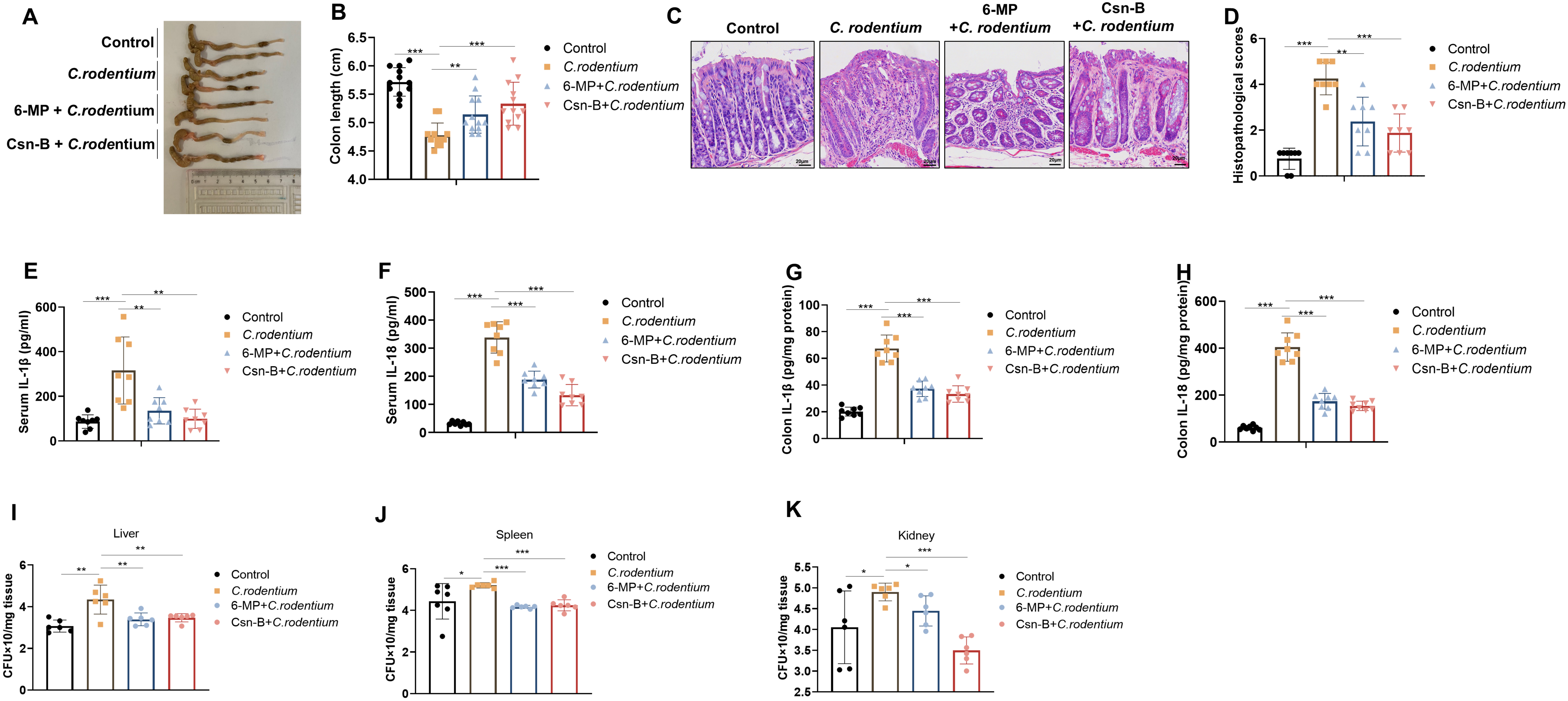

Figure S10. Activating NR4A1 relieves C. rodentium-induced colitis and intestinal infection in mice.

C. rodentium (5×10^9 CFU) was gavaged for once at day 0, at day 1 6-MP (10 mg/kg) and Csn-B (10 mg/kg) were intraperitoneal injected for 7 days mice were sacrificed at day 8 after colitis induction (n = 12/group). (A, B) Images and statistical analysis of colon length. (C, D) Images and histopathological scores of the colon tissues. (E, F) Production of IL-1β and IL-18 in mice serum. (G, H) Production of IL-1β and IL-18 in mice colon tissue. (I-K) The number of *C. rodentium* colonized in the liver, spleen, and kidney (n=6-8/group). Values are expressed as mean ± SD, *P < 0.05.
